# Thermogravimetric Analysis and Kinetic Modeling of the AAEM-Catalyzed Pyrolysis of Woody Biomass

**DOI:** 10.3390/molecules27227662

**Published:** 2022-11-08

**Authors:** Wei Wang, Romain Lemaire, Ammar Bensakhria, Denis Luart

**Affiliations:** 1 Department of Mechanical Engineering, École de Technologie Supérieure, Montreal, QC H3C 1K3, Canada; 2 Centre de Recherche de Royallieu, Université de Technologie de Compiègne, EA 4297-TIMR, BP20529, 60205 Compiègne, France; 3 École Supérieure de Chimie Organique et Minérale, 1 Rue du Réseau Jean-Marie Buckmaster, 60200 Compiègne, France

**Keywords:** pyrolysis, wood, kinetics, catalyst, alkali and alkaline earth metals

## Abstract

This work analyzes the catalytic effects induced by alkali and alkaline earth metals (AAEMs) on pyrolysis kinetics. To this end, thermogravimetric analyses (TGA) were carried out with raw beech wood and samples impregnated with NaCl, KCl and MgCl_2_ at four heating rates (5, 10, 15 and 30 °C/min). Obtained results showed that AAEM compounds promote the decomposition of biomass by reducing the initial and peak pyrolysis temperatures. More specifically, the catalytic effect of the alkaline earth metal was shown to be stronger than that of alkali metals. To further interpret the obtained trends, a kinetic modeling of measured data was realized using two isoconversional methods (the Ozawa–Flynn–Wall (OFW) and Kissinger–Akahira–Sunose (KAS) models). With a view to identifying a suitable reaction model, model fitting and master plot methods were considered to be coupled with the isoconversional modeling approaches. The 3-D diffusion reaction model has been identified as being well suited to properly simulate the evolution of the conversion degree of each sample as a function of the temperature. Furthermore, the kinetic parameters derived from the present modeling work highlighted significant decreases of the activation energies when impregnating wood with AAEM chlorides, thus corroborating the existence of catalytic effects shifting the decomposition process to lower temperatures. A survey of the speculated pathways allowing to account for the impact of AAEMs on the thermal degradation of woody biomass is eventually proposed to better interpret the trends identified in this work.

## 1. Introduction

Alongside increasing concerns about global warming, biomass, a neutral-carbon fuel, is attracting more and more attention, not only for heat and electricity generation, but also for the production of valuable biochemicals and biomaterials. When being pyrolyzed under an inert atmosphere, the biopolymers composing the lignocellulosic biomass typically decompose into biochar, bio-oil and incondensable biogas [1]. However, the yields and selectivity towards target products (e.g., bio-oil) remain limited. Furthermore, and despite the major progress that has been achieved in the field of biomass pyrolysis, bio-oils issued from the thermal conversion of raw biomass generally suffer from a high oxygen content, viscosity and corrosiveness, together with a low heating value [2]. To overcome these limitations, the implementation of a catalytic treatment represents an interesting option. It indeed allows upgrading obtained pyrolysis products by reducing the yields of undesirable components while improving the properties of the target products [3,4].

Among the catalysts currently considered in the framework of biomass pyrolysis, specific attention has recently been devoted to alkali and alkaline earth metals (AAEMs) due to their low toxicity, affordable price, high catalytic efficiency and non-negligible quantity in raw biomass [5]. AAEM compounds can be directly mixed with biomass through wet impregnation (in situ configuration) or brought in downstream in order to react with the vapors produced during pyrolysis (ex situ configuration) [3]. These convenient catalysts can, moreover, be implemented in a wide range of reactor configurations, including fixed bed [6,7] and fluidized bed [8,9] pyrolyzers. To date, most of the studies undertaken to elucidate the impact of AAEMs on the catalytic conversion of biomass have focused on the analysis of the reaction products to evaluate the efficiency of the different alkali and alkaline earth metals in promoting the formation of some target molecules. For instance, Peng et al. investigated the effect of different alkaline additives (NaOH, KOH, Na_2_CO_3_ and K_2_CO_3_) on the production of phenols during the pyrolysis of lignin [10]. They notably observed that all alkalis promote decarboxylation or decarbonylation reactions as well as the removal of unsaturated alkyl-branched chains from aromatic molecules [10]. Furthermore, they noted that the strong hydroxide alkalis of NaOH and KOH favor the deoxygenation of methoxy groups, thus leading to phenols free of methoxy groups. Zhang et al. then studied the impact of impregnating camphor branch, corn cob and walnut shell with potassium nitrate on the distribution of bio-oils produced from fast pyrolysis in a fluidized bed [9]. They reported that potassium tends to increase the yields of furans and phenols while decreasing the yields of aldehydes esters and sugars. Reaction pathways involving dehydration, ring scission, depolymerization and cracking processes were especially proposed to account for the influence of potassium on biomass pyrolysis. As far as alkaline earth metals are concerned, Veses et al. also noted that calcium-based materials (such as CaO) tend to promote dehydration reactions, thereby decreasing the acidity and oxygen content of the bio-oils issued from the pyrolysis of forest pine wood while increasing their pH and calorific value [11]. As for divalent magnesium cations contained in MgCl_2_, they have proven to enhance the degradation of hemicellulose to form oxygenate molecules and furans [12] while promoting repolymerization reactions, leading to increased char formation and higher molecular weight compounds in bio-oils [13].

Notwithstanding the findings reported in the above literature survey, kinetic analyses dealing with the AAEM-catalyzed pyrolysis of biomass remain relatively rare, as highlighted in [5]. This lack is all the more critical since pyrolysis, as the first step in the thermochemical conversion of biomass, directly influences the nature and the distribution of the released products, and hence, the subsequent reaction stages, as well as the overall fuel conversion rate [1]. Characterizing pyrolysis kinetics is therefore important for the proper design of reactors and for the optimization of industrial facilities. Among existing models, one can cite the model-free methods [14], the distributed activation energy and the chemical percolation devolatilization models (see [15] and references therein) in addition to simulation tools relying on the density functional theory for instance [16]. These models can be roughly classified into two categories, depending on whether they aim at simulating either the mass loss rate of the fuel, the distribution of the pyrolytic products or both. In the context of TGA-based kinetic analyses, model-free methods remain extensively used in assessing kinetic parameters as they represent an accurate route to directly infer activation energies without the need for any initial assumption regarding the reaction model [17].

With this in mind, the present work aims at analyzing and modeling the kinetics related to the AAEM-catalyzed pyrolysis of a woody biomass. To this end, pyrolysis experiments were conducted with raw beechwood and samples catalyzed with three AAEM compounds (NaCl, KCl and MgCl_2_) added by wet impregnation. Non-isothermal thermogravimetric measurements were performed with heating rates ranging from 5 to 30 °C/min. Obtained results were then modeled by means of two isoconversional models (namely, the Ozawa–Flynn–Wall (OFW) and Kissinger–Akahira–Sunose (KAS) models) to ease the interpretation of obtained trends.

After introducing the experimental methodology together with the theoretical background underlying the OFW and KAS modeling approaches in Section 2, measured data will be presented and commented on in Section 3. Results issued from the kinetic modeling calculations will notably be detailed therein before being compared with experimental results. In a bid to better interpret observed trends as far as the impact of AAEMs is concerned, Section 4 will finally propose a summary of the main mechanisms at play during the AAEM-catalyzed pyrolysis of biomass.

## 2. Methodology

### 2.1. Feedstocks and Sample Preparation

The proximate and ultimate analyses of the beech wood used herein are provided in Table 1.

**Figure 1:**
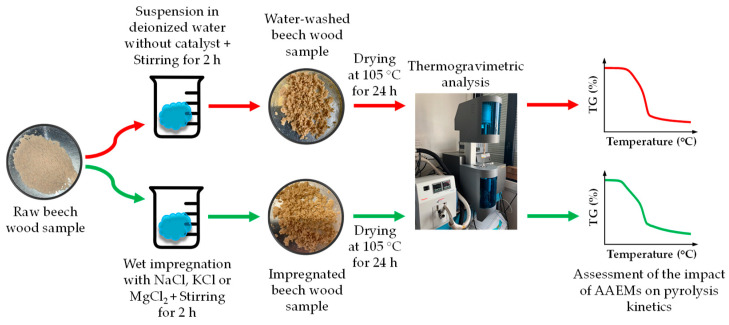
Diagram of sample preparation and analysis procedures.

Three AAEM compounds, namely, NaCl, KCl and MgCl_2_, were selected to be added to beech wood. Samples were prepared by wet impregnation to favor the interaction between the cations and the biomass (see Figure 1). Beech wood samples were thoroughly impregnated in solutions containing exactly the same amount of cation for each AAEM chloride. 4 g of biomass was typically put into prepared NaCl, KCl and MgCl_2_ solutions whose concentration of metal cations had been set to 5 g/L for each catalyst. The mixtures were stirred by means of a magnetic stirrer for two hours, as in [18]. Impregnated samples were then filtered to eliminate the extra cations and anions remaining in the solution. As for the control sample consisting of the raw biomass, it was suspended in deionized water for the same duration and within the same stirring conditions to exclude the effects of water washing. Finally, all the samples were dried in an oven at 105 °C [19,20,21] for 24 h to get rid of the extra free water.

### 2.2. Experimental Apparatus and Procedure

Non-isothermal pyrolysis tests were performed using a SETARAM SETSYS Evolution thermogravimetric analyzer (TGA). Four heating rates (5, 10, 15 and 30 °C/min) were used to heat the samples from room temperature up to 950 °C, with a plateau at 105 °C, for 20 min to ensure the elimination of free water. To perform all the TGA tests, 10 mg of sample was put in alumina crucibles. An inert atmosphere was continuously maintained around heated samples by means of a 100 mL/min nitrogen flow. Three tests were performed for each sample and operating condition. It should therefore be considered that all the mass loss profiles presented in the following and used for the modeling work presented in Section 3 are based on averaged data. Following [22,23], only the section below 700 °C (which represents the main part of the mass loss process) was taken into account for the calculations presented hereafter to get rid of the possible measurement noise encompassing the data recorded at the highest temperatures. To conclude, the conversion degree (α) at any given time (t) was calculated from the initial (i) and final (f) residual masses (noted ‘TG’ and expressed in wt%) based on Equation (1):

(1) $$\alpha =\frac{{\mathrm{TG}}_{i}-{\mathrm{TG}}_{t}}{{\mathrm{TG}}_{i}-{\mathrm{TG}}_{f}}$$

where the conversion degrees are set to 0% and 100% for the initial (106 °C) and final (700 °C) measurement points, respectively.

### 2.3. Kinetic Modeling

#### 2.3.1. Isoconversional Models

The variation of the fuel conversion degree α as a function of the temperature T (expressed in K) during TGA experiments can be expressed using an Arrhenius equation of the type:

(2) $$\frac{d\alpha }{\mathrm{dT}}=\frac{A}{\beta }\times \exp\left(-\frac{E_{a}}{R\times T}\right)\times f_{\left(\alpha \right)}$$

where A is the pre-exponential factor (expressed in s^−1^), β is the heating rate, $E_{a}$ stands for the activation energy (in kJ/mol), R is the gas constant (equal to 8.314 J/(mol·K)), while $f_{\left(\alpha \right)}$ denotes the reaction model. By integrating both sides of Equation (2) while assuming that the initial temperature and conversion degree are equal to zero, the integral form of the reaction model $g_{\left(\alpha \right)}$ can be expressed as follows:

(3) $$g_{\left(\alpha \right)}=\int_{0}^{\alpha }\frac{d\alpha }{f_{\left(\alpha \right)}}=\frac{A}{\beta }\times \int_{T_{0}}^{T}\exp\left(-\frac{E_{a}}{R\times T}\right)\mathrm{dT}≅\frac{A}{\beta }\times \int_{0}^{T}\exp\left(-\frac{E_{a}}{R\times T}\right)\mathrm{dT}$$

The term on the right-hand side of Equation (3) corresponds to an exponential integral which has no exact solution (in closed form). An algebraic approximation of this term must therefore be used to enable the calculation [24,25]. By replacing dT by du in the integral (with $u=\frac{E_{a}}{R\times R}$), the right-hand term of Equation (3) can thus be converted into the form:

(4) $$g_{\left(\alpha \right)}=\frac{A}{\beta }\times \int_{0}^{T}\exp\left(-\frac{E_{a}}{R\times T}\right)\mathrm{dT}=\frac{A}{\beta }\times \int_{\infty }^{u}(-e^{-u}\times \frac{E_{a}}{{R\times u}^{2}})\mathrm{du}=\frac{{A\times E}_{a}}{\beta \times R}\times \int_{u}^{+\infty }(\frac{e^{-u}}{u^{2}})\mathrm{du}=\frac{{A\times E}_{a}}{\beta \times R}\times p\left(u\right)$$

such that:

(5) $$\ln\left[g_{\left(\alpha \right)}\right]=\ln(\frac{{A\times E}_{a}}{\beta \times R})+\ln\left[p\left(u\right)\right]$$

where $p\left(u\right)$ is an exponential integral that does not have an analytical solution. Based on Doyle’s assumption [26], the function $p\left(u\right)$ can be converted into a series allowing deriving the expressions of the OFW and KAS models detailed below.

As for the OFW integral isoconversional model [27,28], it consists of plotting the evolution of $\ln\left(\beta_{i}\right)$ as a function of $-\frac{1}{T}$ for different heating rates $\beta_{i}$ to obtain linearized straight lines whose slopes allow deriving activation energy values for each conversion degree α (see Equation (6)).

(6) $$\ln\left(\beta_{i}\right)=\ln\left(\frac{A_{\alpha }\times E_{a,\alpha }}{R\times g_{\left(\alpha \right)}}\right)-5.331-1.052\times \frac{E_{a,\alpha }}{R\times T}$$

Regarding the values of $A_{\alpha }$, they can be inferred using Equation (7) once the reaction model $g_{\left(\alpha \right)}$ and the activation energies are determined [27]:

(7) $$A_{\alpha }=\frac{R\times \exp\left(b+5.331\right)\times g_{\left(\alpha \right)}}{E_{a,\alpha }}$$

where b is the intercept of the linearized straight lines.

As far as the KAS model is concerned, it consists of plotting straight lines depicting the evolution of $\ln\left(\frac{\beta_{i}}{T^{2}}\right)$ as a function of $-\frac{1}{T}$ for different heating rates $\beta_{i}$. One can then infer the values of the activation energy ($E_{a,\alpha }$) and pre-exponential factor ($A_{\alpha }$) for any conversion degree α by means of the slopes and intercepts of the so-obtained straight lines (see Equations (8) and (9), respectively).

(8) $$\ln\left(\frac{\beta_{i}}{T^{2}}\right)=\ln\left(\frac{A_{\alpha }\times R}{E_{a,\alpha }\times g_{\left(\alpha \right)}}\right)-\frac{E_{a,\alpha }}{R\times T}$$

(9) $$A_{\alpha }=\frac{E_{a,\alpha }\times \exp\left(b\right)\times g_{\left(\alpha \right)}}{R}$$

#### 2.3.2. Methodology Allowing to Identify a Suitable Reaction Model

With a view to selecting a proper reaction model, two possible routes are summarized in the review by Wang et al. [29]. The first solution relies on the selection of the reaction model by comparing the values of the activation energy derived from model-free (i.e., isoconversional) and model fitting methods (see the ‘Model Fitting Approach’ subsection below). Assuming that the isoconversional methods allow assessing more accurate activation energies, these $E_{a}$ values should therefore be considered in the model fitting procedure in order to select an appropriate reaction model allowing to derive similar activation energies [30,31,32,33]. The second route concerns the use of the master plot method (see the ‘Master Plot’ subsection), which allows identifying the most suitable reaction model by comparing experimental curves to some pre-established theoretical ones [33,34,35,36].

##### Model Fitting Approach

The data fitting method selected herein consists of the Šatava–Šesták model [37], which is commonly used in studies dealing with the thermal decomposition of solid materials [38,39,40,41,42,43]. By plotting the evolution of $\log\left(g_{\left(\alpha \right)}\right)$ as a function of $\frac{1}{T}$ for different $g_{\left(\alpha \right)}$ formulations, straight lines can be obtained (see Equation (10)) with their slopes allowing to derive activation energy values. These latter can then be used to identify a proper reaction model that allows obtaining $E_{a}$ values matching those assessed by means of model-free methods. Finally, the $\log\left(\frac{A\times E_{a}}{\beta \times R}\right)$ term enables inferring the value of the pre-exponential factor A:

(10) $$\log\left(g_{\left(\alpha \right)}\right)=\log\left(\frac{A\times E_{a}}{\beta \times R}\right)-2.315-0.4567\times \frac{E_{a}}{R\times T}$$

In the present work, 10 different reaction models were tested, namely, F1, F2, F3, D2, D3, R2, R3, A2, A3 and A4 [44,45].

##### Master Plot

To ease the identification of a suitable reaction model, Sánchez-Jiménez et al. proposed a calculation procedure consisting of transforming measured data into an experimental master plot that does not depend on experimental conditions [46]. This master plot is then compared with theoretical master plots obtained by using different reaction models. By means of a simple graphical procedure, the most suited kinetic model can be identified. In this process, one must first introduce a so-called generalized time, θ, whose derivative over t can be written as:

(11) $$\frac{d\theta }{\mathrm{dt}}=\exp\left(-\frac{E_{a}}{R\times T}\right)$$

By combining the general expression of dα/dt (i.e.,$\;\frac{d\alpha }{\mathrm{dt}}=A\times \exp\left(-\frac{E_{a}}{R\times T}\right)\times f_{\left(\alpha \right)}$) with dθ/dt (see Equation (11)), dα/dθ can be expressed in the form:

(12) $$\frac{d\alpha }{d\theta }=\frac{A\times \exp\left(-\frac{E_{a}}{R\times T}\right)\times f_{\left(\alpha \right)}}{\exp\left(-\frac{E_{a}}{R\times T}\right)}=A\times f_{\left(\alpha \right)}$$

For a single-step process, the expression of the reaction model is invariable. Using a reference point at α = 50%, one obtains:

(13) $${\frac{{\left(\frac{d\alpha }{d\theta }\right)}_{\alpha }}{{\left(\frac{d\alpha }{d\theta }\right)}_{50\%}}}_{\mathrm{theo}}=\frac{f_{\left(\alpha \right)}}{f_{\left(50\%\right)}}$$

where the generalized reaction rate can be expressed as follows:

(14) $$\frac{d\alpha }{d\theta }=\frac{d\alpha }{\mathrm{dt}}\times \exp\left(\frac{E_{a}}{R\times T}\right)$$

so that the relationship between the generalized reaction rate and the experimental data are expressed as:

(15) $${\frac{{\left(\frac{d\alpha }{d\theta }\right)}_{\alpha }}{{\left(\frac{d\alpha }{d\theta }\right)}_{50\%}}}_{\exp}=\frac{{\left(\frac{d\alpha }{\mathrm{dt}}\right)}_{\alpha }}{{\left(\frac{d\alpha }{\mathrm{dt}}\right)}_{50\%}}\times \frac{\exp\left(\frac{E_{a}}{R\times T}\right)}{\exp\left(\frac{E_{a}}{{R\times T}_{50\%}}\right)}$$

By comparing theoretical and experimental curves (whose plotting requires the estimation of $E_{a}$ through isoconversional methods under non-isothermal conditions), the $f_{\left(\alpha \right)}$ formulation leading to the best match between calculated and measured data can be identified.

## 3. Results and Discussion

Thermogravimetric analysis results will first be presented and discussed in Section 3.1 before being modeled by means of the OFW and KAS isoconversional approaches in Section 3.2.

### 3.1. Experimental Characterization of the Pyrolysis Behavior of Raw and Impregnated Beech Wood Samples

**Figure 2:**
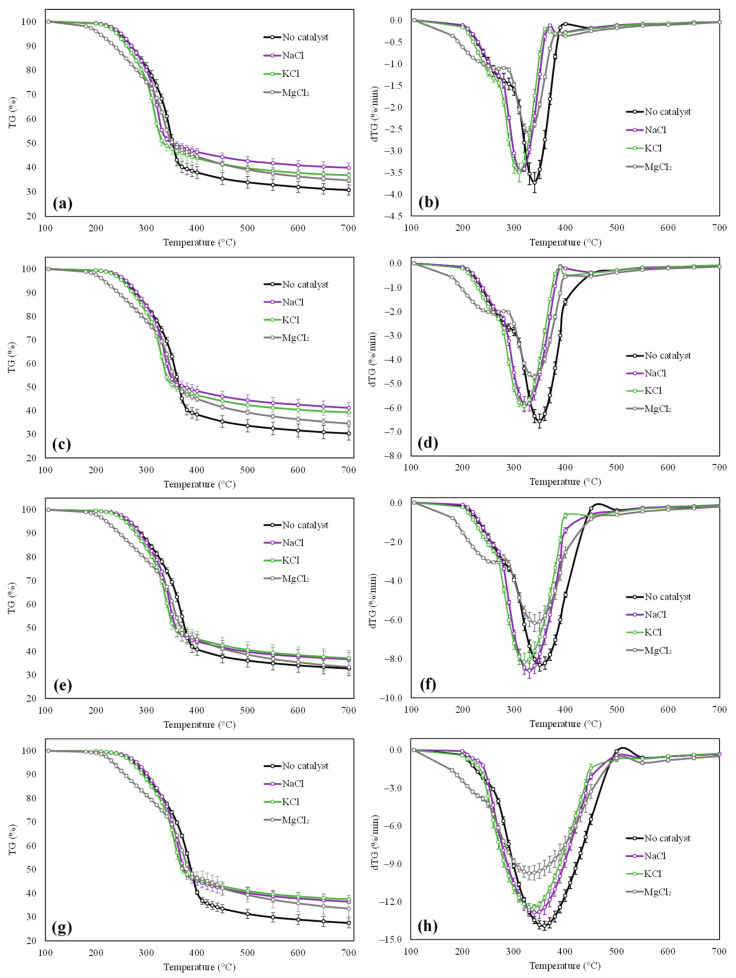
Evolution of mass loss noted ‘TG’ (**a**,**c**,**e**,**g**) and derivate mass loss rate noted ‘dTG’ (**b**,**d**,**f**,**h**) as a function of the temperature for heating rates of 5 °C/min (**a**,**b**), 10 °C/min (**c**,**d**), 15 °C/min (**e**,**f**) and 30 °C/min (**g**,**h**).

Results issued from the TGA analyses conducted with raw and impregnated beech wood samples are detailed in Figure 2. Curves depicting variations of the mass loss (TG) and mass loss rate (dTG) as a function of the temperature are notably reported therein for heating rates of 5, 10, 15 and 30 °C/min. As explained in Section 2.2, three tests were performed for each sample and operating condition. Error bars plotted in Figure 2 consequently account for the dispersion of the experimental data around the mean values.

Based on obtained TG and dTG curves, it can first be noted that the pyrolysis behaviors of the four tested samples significantly diverge from one another regardless of the heating rate. More particularly, the temperatures at which pyrolysis takes place appear to be higher for the raw biomass than for the impregnated samples.

To better highlight and discuss the observed discrepancies, Table 2 reports the characteristic pyrolysis temperatures. This includes the initial (T_i_) and final (T_f_) decomposition temperatures estimated for conversion degrees of 10 and 90%, respectively, as well as the peak temperature (T_p_) assessed at the peak mass loss rate.

Based on the data gathered in Figure 2 and Table 2, it can be noted that the beech wood starts to decompose at temperatures between 257.5 and 286.4 °C for 5 °C/min < β < 30 °C/min.

As shown in Figure 2b,d,f,h, the dTG curves reach a peak for temperatures between 340 and 361 °C (corresponding to the decomposition of cellulose), with shoulders located on the left for temperatures of around 250–300 °C (denoting the degradation of hemicellulose) [47]. The mass loss rates then decrease to plateau above 412–432 °C, depending on the considered heating rate. As far as impregnated samples are concerned, plotted TG and dTG curves exhibit more or less reduced T_i_ and T_p_ values, which is consistent with the role played by alkali and alkaline earth metals on the reduction of the pyrolysis temperatures [13,20,48,49,50,51,52,53]. AAEM cations are indeed likely to cleave chemical bonds during the impregnation process, and hence favor the decomposition of the biomass structure, especially at the beginning of the pyrolysis process (i.e., for relatively low temperatures). It is interesting here to note that the tested alkaline earth metal (magnesium) seems to exhibit a higher catalytic effect than the two alkali metals (sodium and potassium), as exemplified by the greater T_i_ reductions observed with MgCl_2_ regardless of the heating rate. Impregnating beech wood with magnesium chloride indeed allows reducing the initial pyrolysis temperatures by 43 °C on average when considering all β values as compared to the T_i_ values measured for the raw biomass sample. On the other hand, potassium only induces a mean T_i_ drop of 10 °C against 1.6 °C for sodium, which mainly shows a catalytic effect for temperatures higher than 300 °C.

It is noteworthy that these trends are actually in line with those identified in previous investigations dealing with the impact of AAEMs on the pyrolysis of woody biomass components. Yu et al. indeed reported that magnesium was able to lower the onset temperature of cellulose pyrolysis [51]. Gao et al. and Leng et al. also reported that T_i_ values were much more reduced when using alkaline earth metals such as calcium and magnesium instead of potassium to catalyze the pyrolysis of rice straw and cellulose, respectively [52,53].

As for the peak temperatures, similar trends are observed for all catalysts. T_p_ indeed decreases by 22, 29 and 15 °C on average for the samples impregnated with NaCl, KCl and MgCl_2_, respectively (see Table 2). It is interesting to observe that the effect of sodium and potassium on T_p_ is much more significant than on T_i_. Furthermore, the reduction of T_p_ induced by the use of magnesium chloride turns out to be the lowest, which contrasts with the fact that such a catalyst was previously shown to induce the highest T_i_ drop. Looking at the residual masses measured at 700 °C (noted ‘TG_700°C_‘ in Table 2), it can be noted that these latter increase (from +3.7 for MgCl_2_ to +8.2 wt% for NaCl on average) when impregnating beech wood with AAEMs. Mean TG_700°C_ values calculated based on all heating rates indeed pass from 30.4 wt% for the raw biomass to 38.6 wt%, 37.7 wt% and 34.1 wt% when using sodium-, potassium- and magnesium-based catalysts, respectively. Such a trend, which here again, is in line with the observations made in previous studies [13,21,49,51,54,55,56,57], can be traced to an enhanced char formation and/or to recombination reactions occurring at the last stage of the pyrolysis process when the temperature exceeds 350 °C. All the results presented within this section will, however, be further discussed in Section 4, which deals with the description of the main mechanisms underlying the AAEM-catalyzed pyrolysis of biomass components.

### 3.2. Kinetic Analysis

As illustrated in Section 2.3.1, isoconversional models allow inferring $E_{a}$ values without requiring any a priori assumption regarding the selection of the reaction model. As such, these methods generally lead to the derivation of consistent activation energies [29,58], which can be used as inputs in subsequent calculation stages to identify suitable reaction models and assess proper values of pre-exponential factors. This type of calculation procedure implemented in [30] has since been widely used and/or recommended for the modeling of data issued from non-isothermal pyrolysis experiments [29,31,38,41,59,60,61,62]. Following a similar approach, OFW and KAS models were thus implemented herein (see Section 3.2.1) before being coupled with Šatava–Šesták and master plot calculation procedures (see Section 3.2.2).

#### 3.2.1. Isoconversional Modeling of TGA Results

**Figure 3:**
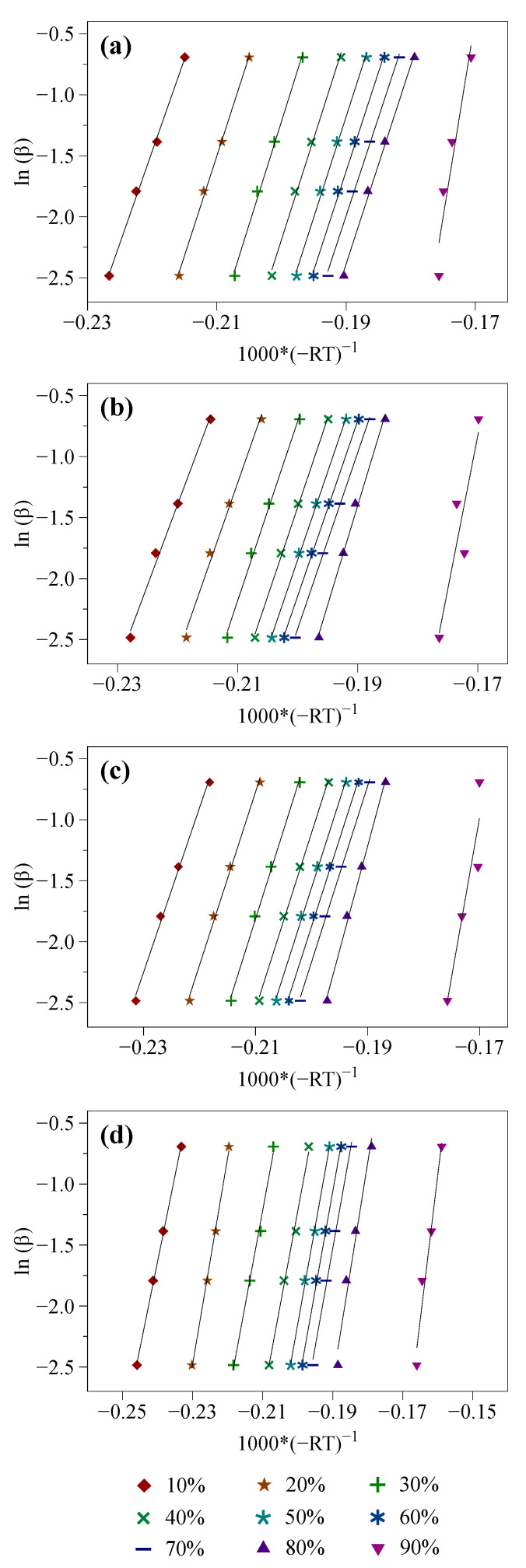
Linear fitting obtained with the OFW model (i.e., plots of $\ln\left(\beta \right)$ as a function of $-\frac{1000}{R\times T}$ for conversion degrees between 10 and 90%) for (**a**) wood, (**b**) wood + NaCl, (**c**) wood + KCl and (**d**) wood + MgCl_2_.

**Figure 4:**
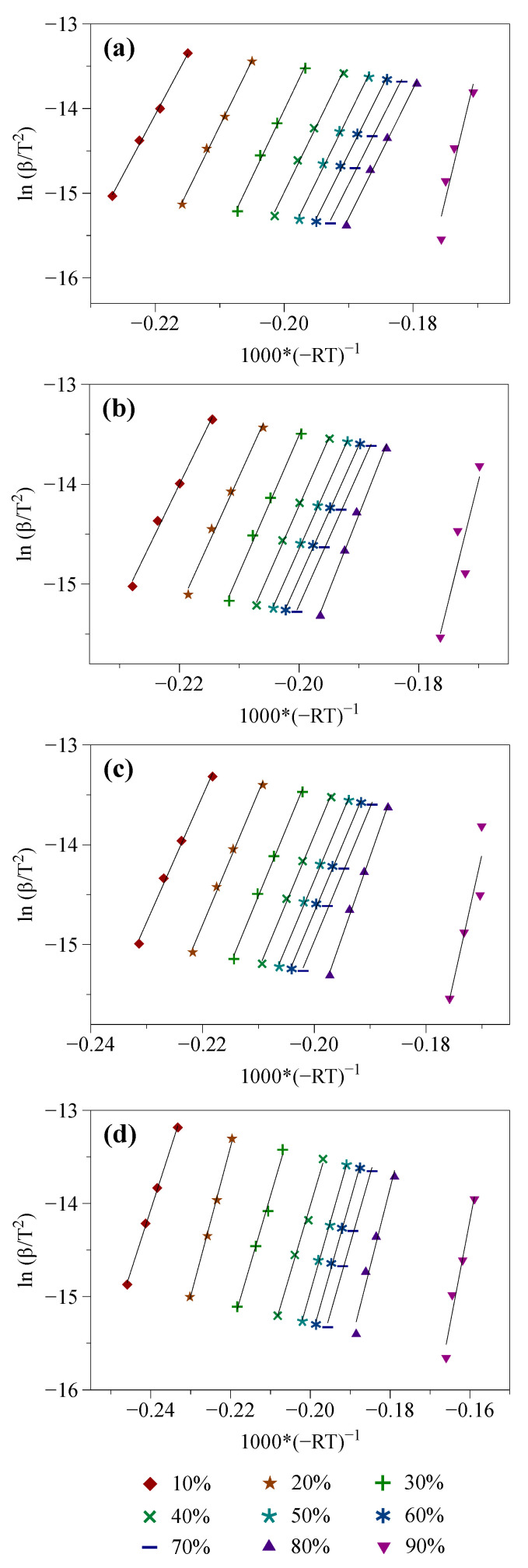
Linear fitting obtained with the KAS model (i.e., plots of $\ln\left(\frac{\beta }{T^{2}}\right)$ as a function of $-\frac{1000}{R\times T}$ for conversion degrees between 10 and 90%) for (**a**) wood, (**b**) wood + NaCl, (**c**) wood + KCl and (**d**) wood + MgCl_2_.

The OFW and KAS isoconversional models were implemented following the calculation procedures described in Section 2.3.1. Doing so led to the obtention of the linearized straight lines depicted in Figure 3 and Figure 4, from which the values of the α-dependent activation energy reported in Table 3 were inferred.

As previously mentioned (see Section 2.2), results issued from three tests were averaged for each sample and operating condition to mitigate the deviations possibly observed from test to test due to measurement noise and uncertainties. As such, it was possible to obtain very good linear correlations, as shown in Figure 3 and Figure 4, and evidenced by the high coefficients of determination reported in Table 3 (R^2^ ≈ 0.985 on average). As can be seen, for each 10% step of the conversion degree, identical activation energies are obtained with the OFW and KAS models (see Table 3). The mean relative standard deviation between the $E_{a}$ values assessed using both methods is indeed of only 1.51%. Furthermore, and overall, the higher the conversion degree, the higher the activation energy, as also noted in [58,63,64,65,66,67], which is consistent with what may have been expected since the species emitted at high temperatures typically require more energy to be released.

When analyzing the results gathered in Table 3 in greater detail, it can be noted that the activation energies assessed for raw biomass with the OFW and KAS models are quite constant for 20% ≤ α ≤ 80%, with values ranging from 153.0 to 160.5 kJ/mol with the OFW model and from 150.3 to 159 kJ/mol with the KAS one. On the other hand, the activation energy increases significantly, up to around 310 kJ/mol, when the conversion degree reaches 90%, which may be related to the decomposition of lignin that contains more rigid carbon-carbon linkages requiring more energy to be cleaved [29]. As for the samples impregnated with NaCl, KCl and MgCl_2_, the range of $E_{a}$ values estimated for 20% ≤ α ≤ 70% is also relatively narrow (i.e., between ~132 and ~140 kJ/mol for NaCl and KCl compared to values between ~145 and ~161 kJ/mol for MgCl_2_). The activation energies then increase for higher conversion degree, thus indicating a pyrolysis behavior relatively similar to that described in the case of beech wood. Another interesting feature emerging from the tests performed with NaCl, KCl and MgCl_2_ concerns the global reduction of the $E_{a}$ values resulting from the impregnation of beech wood with these AAEM-catalysts. Mean decreases of the activation energies between ~5 and ~19 kJ/mol are indeed observed for 10% ≤ α ≤ 70%. Since the activation energy accounts for the minimum energy required for a reaction to occur, any decrease of $E_{a}$ tends to indicate that less external energy is required to overcome the energy barrier allowing the pyrolysis to take place. As a consequence, the data reported in Table 3 tend to corroborate one of the experimental observations made in Section 3.1 regarding the catalytic effect induced by AAEM compounds, which are able to shift the decomposition of biomass to lower temperatures. Lastly, it is noteworthy that the $E_{a}$ values inferred herein are consistent with those reported in the literature for widely varied biomass sources with values which are generally between 100 and 300 kJ/mol [29]. Nevertheless, interpreting the kinetic features related to the AAEM-catalyzed pyrolysis of beech wood in greater detail requires conducting additional analyses with the view to identifying a suitable reaction model, as detailed in Section 3.2.2.

#### 3.2.2. Identification of a Proper Reaction Model

##### Selection of a Proper $g_{\left(\alpha \right)}$ Formulation through Model-Fitting Calculations

**Figure 5:**
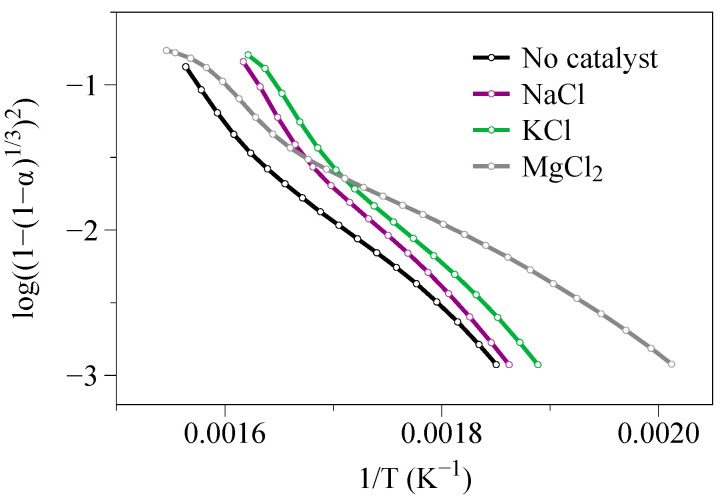
Example of linear data fitting issued from the implementation of the Šatava–Šesták model while considering the D3 reaction model for a heating rate of 10 °C/min.

The Šatava–Šesták model was implemented following the procedure described in Section 2.3.2. Curves depicting the evolution of $\log\left(g_{\left(\alpha \right)}\right)$ as a function of $\frac{1}{T}$ were plotted for 10 reaction models commonly used in the literature. To this end, only the data associated with conversion degrees between 10 and 80% (i.e., those free from potentially important measurement noise) were considered to enable consistent data fitting calculations. Among tested reaction models, the focus was placed on order-based (F_n_), diffusion (D_n_), geometrical contraction (R_n_) and nucleation (A_n_) ones, as listed in Section 2.3.2 [44,45,64]. An example of data fitting based on the results obtained for a heating rate of 10 °C/min with the D3 reaction model is presented in Figure 5. By repeating the same procedure for the 10 reaction models considered herein (i.e., F1, F2, F3, D2, D3, R2, R3, A2, A3 and A4), a series of linearized curves were obtained, with their slopes allowing to infer the values of the apparent activation energy.

**Figure 6:**
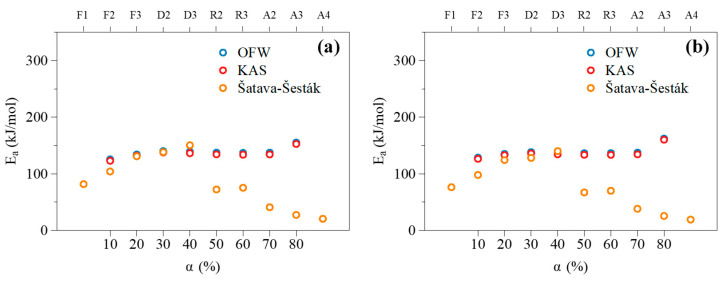
Isoconversional model plot of $E_{a}$ obtained with the OFW, KAS and Šatava–Šesták models in the case of samples impregnated with (**a**) NaCl and (**b**) KCl, as examples.

Although most of the tested models allow obtaining a high coefficient of correlation (0.93 ≤ R^2^ ≤ 0.99), identifying a proper model based solely on R^2^ values may be inadequate, as highlighted by Khawam and Flanagan [30]. According to these authors, the selection of an adapted reaction model should be preferentially achieved based on isoconversional model plots (IMO) of activation energies as a function of α. Following this approach, $E_{a}$ values derived from the OFW and KAS models were compared with those inferred by means of the Šatava–Šesták model integrating the 10 mechanisms listed above. To this end, linear data fittings were performed at each investigated heating rate with all the $g_{\left(\alpha \right)}$ formulations (data not reported herein for brevity). Obtained results then showed that β had no significant influence on the so-inferred $E_{a}$ values. The relative standard deviation between the activation energies assessed at 5, 10, 15 and 30 °C/min was indeed found to be less than 0.6% on average for all the reaction models in the case of the samples impregnated with NaCl, as an example. Mean activation energies were therefore considered, as in [38], and plotted in Figure 6 for comparison purposes. The best suited reaction model can then be identified, and corresponds to that whose implementation in the model fitting procedure allows assessing $E_{a}$ values closest to those derived when using the model-free approaches [30,38].

Obtained plots thus show that order-based (F3) and diffusion (D2 and D3) models allow obtaining activation energies quite close to those inferred using the OFW and KAS models. Nevertheless, the D3 model still appears to be the most suited. Its use indeed leads to the lowest deviations between the $E_{a}$ values inferred by means of the model fitting and isoconversional approaches (~4% compared to 4.7 and 8.8% for the D2 and F3 models, respectively, based on the results reported in Figure 6). Nevertheless, and before drawing a related clear-cut conclusion, the suitability of the D3 model will be verified.

##### Validation of the Selected $g_{\left(\alpha \right)}$ Formulation through Direct Calculations

**Figure 7:**
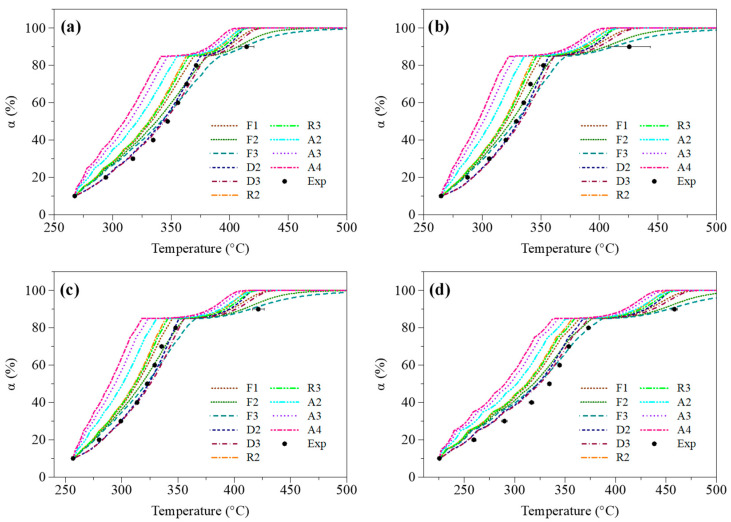
Evolution of α as a function of the temperature for a heating rate of 10 °C/min in the case of (**a**) wood and samples impregnated with (**b**) NaCl, (**c**) KCl and (**d**) MgCl_2_; comparison of experimental data (noted ‘Exp’) with predicted ones obtained from the use of the KAS model integrating 10 different reaction mechanisms.

To corroborate the conclusion drawn above, we carried out complementary calculations aimed at stimulating the evolution of the conversion degree of each sample as a function of the temperature when considering the 10 reaction models used herein. Obtaining such curves, however, required integrating both $E_{a}$ and A in Equation (2). Pre-exponential factors were therefore assessed in addition to the activation energies listed in Table 3. To that end, the ten $g_{\left(\alpha \right)}$ formulations were integrated in Equations (7) and (9) to perform the simulations based on the OFW and KAS models, respectively. Proceeding as such allowed to derive the pre-exponential factors listed in Tables S1–S4, which are provided as Supplementary Materials to this article (see the Supplementary Materials). Introducing these parameters in Equation (2) then led to the obtention of the kinetic profiles depicted in Figure 7, which display the evolution of α as a function of T calculated based on the KAS model for a heating rate of 10 °C/min (data related to the OFW model are reported in Figure S1 of the Supplementary Materials, for their part). To obtain these conversion degree profiles, the temperature values reported in Table 2 for an α of 10% were used as initial reaction temperatures. Furthermore, calculations were carried out by applying the kinetic parameters derived for a given α on a ±5% conversion degree range (e.g., rate constant parameters estimated for α = 20% were kept constant to perform the calculations on the 15% < α < 25% range).

As can be seen by looking at Figure 7, all tested models globally reproduce the overall shape of the measured conversion degree profiles. Some mechanisms (e.g., F2, F3, D2), however, exhibit better predictive capabilities, as is especially the case of the D3 model previously identified as being the most suited. Such a direct calculation procedure thus tends to confirm the relevance of the 3-D diffusion reaction model, as further verified through master plot calculations below.

##### Validation of the Selected $g_{\left(\alpha \right)}$ Formulation through Master Plot Calculations

Following the procedure described in Section 2.3.2, the generalized master plot method proposed by Sánchez-Jiménez et al. was implemented on a range of α for which the rate constant parameters were quite constant. Indeed, and as reiterated in Section 2.3.2, the master plot method is only applicable to single-step processes for which the activation energy does not vary with the conversion degree. As illustrated in Table 3, such a condition is fulfilled when 10% < α < 80% for the different tested samples, which corresponds to the main pyrolysis stage. Measured data were thus transformed into experimental master plots which were compared with theoretical master plots derived from the use of the F1, F2, F3, D2, D3, R2, R3, A2, A3 and A4 reaction models.

**Figure 8:**
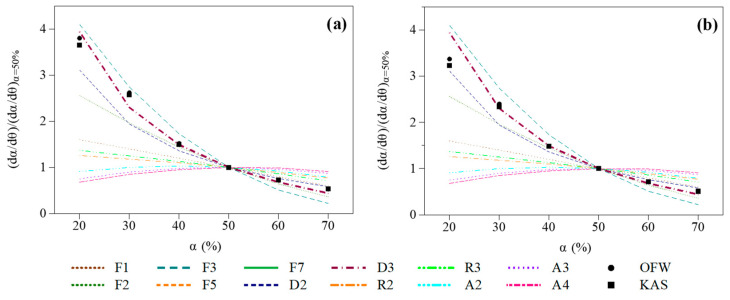
Comparison of experimental (β = 10 °C/min) and theoretical master plots for samples impregnated with (**a**) NaCl and (**b**) KCl.

Figure 8 depicts the results obtained with the samples impregnated with NaCl and KCl, as examples for a heating rate of 10 °C/min. Results depicted therein confirm that the D3 reaction model appears to be the most suited to match experimental points (similar trends being obtained with the other samples). This conclusion is actually consistent with the fact that diffusion models are generally recommended in kinetic analyses aimed at investigating biomass pyrolysis [63,64,67,68,69]. This, moreover, confirms the interest of applying the master plot method to select a proper reaction model, as highlighted in a recent review by Wang et al. focusing on biomass pyrolysis [29].

#### 3.2.3. Simulation of Conversion Degree Profiles

**Figure 9:**
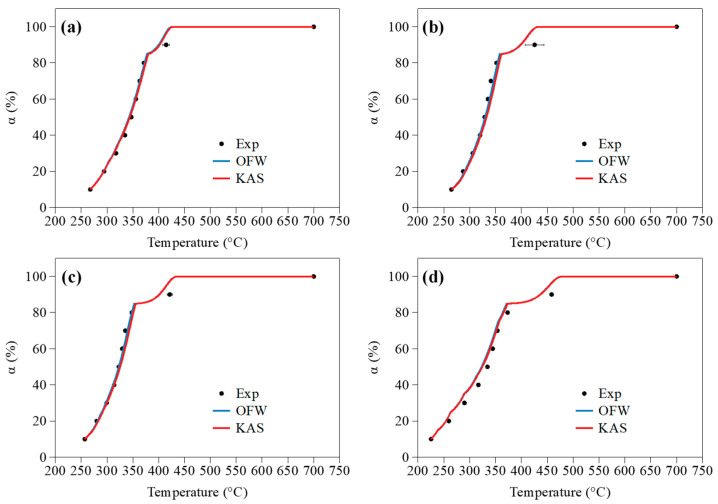
Evolution of α as a function of the temperature for a heating rate of 10 °C/min in the case of (**a**) wood and samples impregnated with (**b**) NaCl, (**c**) KCl and (**d**) MgCl_2_; comparison of experimental data (noted ‘Exp’) with predicted ones obtained from the use of the OFW and KAS models integrating the D3 reaction mechanism.

By integrating the parameters assessed by means of the OFW and KAS models in Equation (2) while considering the so-identified D3 mechanism, one obtains the conversion degree profiles depicted in Figure 9 for a heating rate of 10 °C/min, as an example (profiles related to β values of 5, 15 and 30 °C/min are reported in Figures S2–S4 of the Supplementary Materials).

As can be seen, simulated data satisfactorily reproduce their experimental counterparts regardless of the considered sample. As such, the results depicted in Figure 9 tend to corroborate the consistency of the rate constant parameters and reaction mechanism assessed through this modeling work. To conclude the analysis of the catalytic effect induced by AAEMs on the pyrolysis kinetics of beechwood, the rate constant ($k=A\times \exp\left(-E_{a}/(R\times T)\right)$) of each sample was calculated for temperatures between 250 and 400 °C, which corresponds to the main pyrolysis stage. Here again, the D3 model was considered together with mean apparent rate constant parameters assessed by means of the KAS model for 10% < α < 80% (similar trends are obtained for the OFW model). It is then noteworthy that the rate constant values are higher for impregnated samples. As an example, the rate constants calculated for pure wood and samples impregnated with NaCl, KCl and MgCl_2_ reach values of 1.83 × 10^−4^ s^−1^, 2.92 × 10^−4^ s^−1^, 3.83 × 10^−4^ s^−1^ and 4.26 × 10^−3^ s^−1^, respectively, for a temperature of 325 °C (i.e., 598.15 K). Furthermore, the ability of the tested AAEM chlorides to promote the pyrolysis follows the sequence: MgCl_2_ > KCl > NaCl, which is in line with the experimental observations made in Section 3.1. The modeling work realized herein thus allowed deriving kinetic parameters suitable for experimentally reproducing monitored conversion degree profiles while corroborating, from a kinetic perspective, the higher catalytic efficiency of the tested alkaline earth metal over alkali metals. The mechanisms likely to explain such trends will, however, be discussed further in detail in Section 4.

## 4. Summary of the Mechanisms Underlying the Catalytic Impact of AAEMs on Biomass Pyrolysis

To better understand the observations made in Section 3 regarding the effects of alkali and alkaline earth metals on the thermal degradation of beech wood, this section aims at reviewing the major mechanisms likely to influence the AAEM-catalyzed pyrolysis of biomass. Actually, there are essentially two routes allowing to account for the catalytic effects induced by AAEMs [1,5].

First, alkali and alkaline earth metals have been demonstrated to promote the cleavage of intra- and inter-molecular bonds (i.e., glycosidic and hydrogen bonds) in biomass. They, moreover, favor the cracking of primary volatiles, thus enhancing the yields of light oxygenated compounds and incondensable gaseous species while contributing to limiting the formation of levoglucosan. In that respect, Zhao and Li notably showed that small sodium cations were likely to pierce through rice husk textures to act on biopolymers (i.e., cellulose, hemicellulose and lignin), thus changing the pyrolysis reaction pathways and favoring biomass degradation [70]. More specifically, Na^+^ cations have been proven to act on cellulose through hemolytic cleavage in pyranose rings and heterocyclic cleavage of glycosidic linkage while promoting ring scission, isomerization, dehydration, decarbonylation and/or decarboxylation reactions, which promote the formation of smaller furans and alcohols [48,70]. Besides, sodium cations can also act on the branched-chain structure of hemicellulose to ease depolymerization, ring scission, dehydration as well as rearrangement reactions. Finally, NaCl can enhance the decomposition of lignin by facilitating the chemical bonds of the lignin structure to undergo dehydration, protonation, aromatization and rearrangement reactions. All these processes therefore contribute to favor biomass degradation, which is consistent with the experimental observations and kinetic analyses made throughout the present work. As far as potassium chloride is concerned, Leng et al. showed that such a catalyst was likely to promote the cleavage of glycosidic bonds and the scission of pyran rings directly by homolytic reaction to form low molecular weight species during the fast pyrolysis of cellulose [53]. Furthermore, Safar et al. noted that the amount of intermolecular bonds of cellulose was reduced when impregnating woody biomass with potassium cations, hence decreasing the crystallinity of this biopolymer and increasing the biomass reactivity during pyrolysis [21]. As for magnesium, it has been shown to enhance the degradation of hemicellulose, which results in the formation of furans, while contributing to the initial dehydration/decomposition of cellulose [12,13,71]. All these reaction pathways are therefore likely to explain the decreases in the pyrolysis temperatures and activation energies highlighted in Section 3.

Secondly, AAEMs can play an important catalytic role as bridges linking adjacent oxygenated functional groups. As such, AAEMs are thus likely to promote recombination reactions favoring the formation of char. Hwang et al. notably reported in their study on the pyrolysis of MgCl_2_-impregnated yellow poplar that magnesium could enhance the repolymerization of the volatile molecules released, which would increase the average molecular weight, the viscosity and the solid content of the produced bio-oil despite its higher water content [13]. Such a phenomenon, here again, is well in line with the observations made based on the TG curves related to the AAEM-impregnated samples herein, which exhibited higher residual masses at 700 °C than in the case of raw wood regardless of the considered heating rate.

## 5. Conclusions

The pyrolysis of raw beech wood and samples impregnated with three AAEM catalysts (NaCl, KCl and MgCl_2_) was investigated at four heating rates by means of thermogravimetric analyses. Obtained results showed that the tested alkali and alkaline earth metals promote biomass decomposition by reducing the initial and peak pyrolysis temperatures. The alkaline earth metal (magnesium) was, moreover, found to exhibit a higher catalytic effect than alkali metals (sodium and potassium), as exemplified by its higher ability to reduce initial decomposition temperatures. A comprehensive kinetic analysis of measured data was then realized using two model-free methods (OFW and KAS models). In terms of highlights, the OFW and KAS models led to inferring very similar activation energies, which were, moreover, found to be lower for the samples impregnated with NaCl, KCl and MgCl_2_, thus corroborating the existence of a catalytic effect when such AAEM compounds are added to beech wood. Coupling the above-mentioned isoconversional models with model fitting and master plot approaches allowed to identify the D3 mechanism as being well suited to reproduce experimentally monitored data. While they allowed to simulate the evolution of the conversion degree of each sample as a function of the temperature regardless of the heating rate, the kinetic parameters inferred within this work also led to an illustration of increases of the pyrolysis rate constants when impregnating biomass with AAEMs.

To conclude, the main mechanisms at play during the catalytic pyrolysis of biomass (i.e., the enhanced cleavage of chemical bonds in biomass and the promotion of char formation reactions) have been discussed herein in a bid to interpret the observations made throughout this study. The trends depicted herein thus allowed to corroborate some speculated pathways proposed to account for the impact of AAEMs on the thermal degradation of woody biomass. Furthermore, obtained results also confirmed the interest of coupling varied modeling approaches to contribute to elucidating the fundamentals of AAEM-catalyzed pyrolysis, noting that complementary simulation tools including refined global kinetic schemes as well as phenomenological models would benefit from being considered in complementary studies, thus paving the way for future works to be undertaken.

## Figures and Tables

**Table 1:** Proximate and ultimate analyses of the tested beech wood sample.

Sample	Proximate Analysis			Ultimate Analysis				
	Fixed Carbon (wt%, db ^†^)	Volatiles (wt%, db)	Ash (wt%, db)	C (wt%, daf ^‡^)	H (wt%, daf)	O * (wt%, daf)	N (wt%, daf)	S (wt%, daf)
Beech wood	14.36	84.48	1.16	51.6	5.4	43.0	-	-

^†^ db: dry basis; ^‡^ daf: dry ash-free basis; * calculated by difference.

**Table 2:** Characteristic decomposition temperatures, maximal mass loss rates and residual masses at 700 °C for raw and impregnated beech wood samples for β values of 5, 10, 15 and 30 °C/min.

β	5 °C/min					10 °C/min				
Sample	T_i_ (°C)	T_f_ (°C)	T_p_ (°C)	dTG_max_ (%/min)	TG_700°C_ (wt%)	T_i_ (°C)	T_f_ (°C)	T_p_ (°C)	dTG_max_ (%/min)	TG_700°C_ (wt%)
Wood	257.5 (±0.9)	411.9 (±37.1)	340 (±2)	−3.74 (±0.23)	30.9 (±2.1)	267.5 (±0.2)	414.3 (±6.5)	350 (±1)	−6.55 (±0.29)	30.4 (±2.8)
Wood + NaCl	254.7 (±0.6)	408.8 (±11.0)	315 (±1)	−3.49 (±0.04)	40.0 (±2.0)	264.7 (±1.1)	425.5 (±18.0)	325 (±2)	−5.88 (±0.31)	41.2 (±2.3)
Wood + KCl	246.7 (±0.8)	411.1 (±5.0)	309 (±1)	−3.51 (±0.21)	36.9 (±2.8)	256.8 (±0.5)	421.4 (±5.1)	320 (±3)	−5.92 (±0.19)	39.3 (±1.7)
Wood + MgCl_2_	216.0 (±5.1)	451.6 (±3.4)	329(±0)	−2.59 (±0.08)	34.9 (±1.0)	225.4 (±1.6)	458.2 (±3.1)	340 (±1)	−4.72 (±0.11)	34.5 (±1.4)
** β **	**15 °C/min**					**30 °C/min**				
**Sample**	**T_i_** **(°C)**	**T_f_** **(°C)**	**T_p_** **(°C)**	**dTG_max_ (%/min)**	**TG_700°C_ (wt%)**	**T_i_** **(°C)**	**T_f_** **(°C)**	**T_p_** **(°C)**	**dTG_max_ (%/min)**	**TG_700°C_ (wt%)**
Wood	275.5 (±2.4)	419.7 (±10.3)	353 (±1)	−8.32 (±0.32)	32.7 (±3.1)	286.4 (±2.6)	431.6 (±3.3)	361 (±3)	−13.9 (±0.4)	27.6 (±2.1)
Wood + NaCl	273.7 (±1.4)	420.1 (±5.8)	329 (±2)	−8.61 (±0.39)	36.6 (±2.9)	287.6 (±1.5)	435.0 (±5.1)	344 (±1)	−12.9 (±0.5)	36.5 (±2.7)
Wood + KCl	264.4 (±1.0)	433.1 (±11.6)	323 (±1)	−8.15 (±0.45)	37.1 (±3.3)	278.0 (±3.2)	434.2 (±4.9)	337 (±3)	−12.5 (±0.3)	37.5 (±1.6)
Wood + MgCl_2_	231.4 (±1.1)	470.2 (±21.7)	342 (±3)	−6.18 (±0.44)	33.4 (±2.6)	242.6 (±13.4)	484.4 (±55.6)	331 (±5)	−9.7 (±0.5)	33.6 (±3.3)

**Table 3:** Activation energies issued from the implementation of the OFW and KAS models.

α	Wood		Wood + NaCl		Wood + KCl		Wood + MgCl2	
	$E_{a}\;(\mathrm{kJ}/\mathrm{mol})$	R^2^	$E_{a}\;(\mathrm{kJ}/\mathrm{mol})$	R^2^	$E_{a}\;(\mathrm{kJ}/\mathrm{mol})$	R^2^	$E_{a}\;(\mathrm{kJ}/\mathrm{mol})$	R^2^
OFW								
10%	143.7	0.9978	125.4	0.9938	128.6	0.9961	134.4	0.9991
20%	155.8	0.9982	134.0	0.9926	135.1	0.9967	161.0	0.9983
30%	160.5	0.9963	140.0	0.9953	138.2	0.9974	147.6	0.9946
40%	157.9	0.9954	139.0	0.9984	137.1	0.9980	146.9	0.9937
50%	156.5	0.9966	137.3	0.9993	136.3	0.9983	152.3	0.9986
60%	154.4	0.9971	136.8	0.9994	136.3	0.9983	153.2	0.9978
70%	153.0	0.9971	137.2	0.9993	137.3	0.9982	152.7	0.9955
80%	155.0	0.9967	155.0	0.9949	162.1	0.9972	171.2	0.9744
90%	309.5	0.9096	239.0	0.8348	248.5	0.8853	224.7	0.9647
**KAS**								
10%	142.1	0.9976	122.9	0.9927	126.3	0.9954	133.0	0.9990
20%	154.4	0.9979	131.6	0.9914	132.8	0.9962	160.5	0.9981
30%	159.0	0.9958	137.6	0.9945	135.8	0.9969	145.9	0.9939
40%	156.0	0.9947	136.3	0.9981	134.3	0.9977	144.7	0.9929
50%	154.3	0.9961	134.3	0.9991	133.4	0.9980	150.1	0.9984
60%	151.8	0.9966	133.7	0.9993	133.3	0.9979	150.8	0.9974
70%	150.3	0.9966	134.1	0.9992	134.3	0.9979	150.1	0.9947
80%	152.2	0.9962	152.6	0.9941	160.1	0.9967	169.2	0.9709
90%	314.2	0.9034	239.9	0.8211	249.9	0.8757	223.9	0.9607

## Data Availability

The data presented in this study are available on request from the corresponding author.

## Supplementary Materials

The following supporting information can be downloaded at: https://www.mdpi.com/article/10.3390/molecules27227662/s1, Table S1: Kinetic parameters derived from the use of the OFW and KAS approaches for different reaction models—Wood; Table S2: Kinetic parameters derived from the use of the OFW and KAS approaches for different reactions model—Wood + NaCl; Table S3: Kinetic parameters derived from the use of the OFW and KAS approaches for different reaction models—Wood + KCl; Table S4: Kinetic parameters derived from the use of the OFW and KAS approaches for different reaction models—Wood + MgCl_2_; Figure S1: Evolution of α as a function of the temperature for a heating rate of 10 °C/min in the case of (**a**) wood and samples impregnated with (**b**) NaCl, (**c**) KCl and (**d**) MgCl_2_; comparison of experimental data (noted ‘Exp’) with predicted ones obtained from the use of the OFW model integrating 10 different reaction mechanisms; Figure S2: Evolution of α as a function of the temperature for a heating rate of 5 °C/min in the case of (**a**) wood and samples impregnated with (**b**) NaCl, (**c**) KCl and (**d**) MgCl_2_; comparison of experimental data (noted ‘Exp’) with predicted ones obtained from the use of the OFW, KAS models integrating the D3 reaction mechanism; Figure S3: Evolution of α as a function of the temperature for a heating rate of 15 °C/min in the case of (**a**) wood and samples impregnated with (**b**) NaCl, (**c**) KCl and (**d**) MgCl_2_; comparison of experimental data (noted ‘Exp’) with predicted ones obtained from the use of the OFW, KAS models integrating the D3 reaction mechanism; Figure S4: Evolution of α as a function of the temperature for a heating rate of 30 °C/min in the case of (**a**) wood and samples impregnated with (**b**) NaCl, (**c**) KCl and (**d**) MgCl_2_; comparison of experimental data (noted ‘Exp’) with predicted ones obtained from the use of the OFW, KAS models integrating the D3 reaction mechanism.
