# Supplementary material for: Thermogravimetric Analysis and Kinetic Modeling of the AAEM-Catalyzed Pyrolysis of Woody Biomass
Source: Molecules. 2022 Nov 8;27(22):7662. doi: 10.3390/molecules27227662 (PMC9693403; doi:10.3390/molecules27227662)
Supplement: Supplementary file 1 [file molecules-27-07662-s001.zip › molecules-2016738 - supplementary.pdf]

molecules

Article

Thermogravimetric Analysis and Kinetic Modeling of the AAEM-Catalyzed Pyrolysis of Woody Biomass

Wei Wang [1,2], Romain Lemaire [1,*], Ammar Bensakhria [2] and Denis Luart [3]

[1] Department of Mechanical Engineering, École de Technologie Supérieure, Montreal, QC H3C 1K3, Canada;
[2] Centre de Recherche de Royallieu, Université de Technologie de Compiègne, EA 4297-TIMR, BP20529, 60205 Compiègne, France;
[3] École Supérieure de Chimie Organique et Minérale, 1 Rue du Réseau Jean-Marie Buckmaster, 60200 Compiègne, France;
* Correspondence: romain.lemaire@etsmtl.ca; Tel.: +1-5143968727

The present supplementary material includes a series of 4 tables and 4 figures that supplement the data provided in the main article file.

Table S1. Kinetic parameters derived from the use of the OFW and KAS approaches for different reaction models – Wood.

**OFW**											
**α**	**$E_{a,α}$ (kJ/mol)**	**Pre-exponential factor ($A_α$, s^{-1})**									
		**F1**	**F2**	**F3**	**D2**	**D3**	**R2**	**R3**	**A2**	**A3**	**A4**
10%	143.7	8.1E+10	8.5E+10	9.0E+10	4.0E+09	9.1E+08	3.9E+10	2.6E+10	2.5E+11	3.6E+11	4.4E+11
20%	155.8	4.8E+11	5.4E+11	6.1E+11	4.6E+10	1.1E+10	2.3E+11	1.5E+11	1.0E+12	1.3E+12	1.5E+12
30%	160.5	5.3E+11	6.3E+11	7.7E+11	7.4E+10	1.9E+10	2.4E+11	1.7E+11	8.8E+11	1.0E+12	1.1E+12
40%	157.9	1.7E+11	2.2E+11	2.9E+11	3.1E+10	8.1E+09	7.4E+10	5.2E+10	2.4E+11	2.6E+11	2.8E+11
50%	156.5	9.0E+10	1.3E+11	2.0E+11	2.0E+10	5.5E+09	3.8E+10	2.7E+10	1.1E+11	1.2E+11	1.2E+11
60%	154.4	5.0E+10	8.2E+10	1.4E+11	1.3E+10	3.8E+09	2.0E+10	1.4E+10	5.3E+10	5.3E+10	5.4E+10
70%	153.0	3.5E+10	6.8E+10	1.5E+11	9.9E+09	3.2E+09	1.3E+10	9.7E+09	3.2E+10	3.1E+10	3.1E+10
80%	155.0	4.7E+10	1.2E+11	3.5E+11	1.4E+10	5.0E+09	1.6E+10	1.2E+10	3.7E+10	3.4E+10	3.3E+10
90%	309.5	9.6E+21	3.8E+22	2.1E+23	2.8E+21	1.2E+21	2.9E+21	2.2E+21	6.3E+21	5.5E+21	5.2E+21
**KAS**											
**α**	**$E_{a,α}$ (kJ/mol)**	**Pre-exponential factor ($A_α$, s^{-1})**									
		**F1**	**F2**	**F3**	**D2**	**D3**	**R2**	**R3**	**A2**	**A3**	**A4**
10%	142.1	5.3E+10	5.5E+10	5.8E+10	2.6E+09	5.9E+08	2.6E+10	1.7E+10	1.6E+11	2.4E+11	2.8E+11
20%	154.4	3.3E+11	3.7E+11	4.2E+11	3.2E+10	7.7E+09	1.6E+11	1.1E+11	7.1E+11	9.1E+11	1.0E+12
30%	159.0	3.6E+11	4.3E+11	5.2E+11	5.1E+10	1.3E+10	1.6E+11	1.1E+11	6.0E+11	7.1E+11	7.8E+11
40%	156.0	1.0E+11	1.4E+11	1.8E+11	1.9E+10	5.0E+09	4.6E+10	3.2E+10	1.5E+11	1.6E+11	1.7E+11
50%	154.3	5.3E+10	7.6E+10	1.1E+11	1.2E+10	3.2E+09	2.2E+10	1.6E+10	6.3E+10	6.7E+10	6.9E+10
60%	151.8	2.8E+10	4.5E+10	7.9E+10	7.1E+09	2.1E+09	1.1E+10	8.0E+09	2.9E+10	2.9E+10	3.0E+10
70%	150.3	1.9E+10	3.6E+10	7.8E+10	5.2E+09	1.7E+09	7.0E+09	5.1E+09	1.7E+10	1.6E+10	1.6E+10
80%	152.2	2.5E+10	6.1E+10	1.8E+11	7.3E+09	2.6E+09	8.5E+09	6.4E+09	1.9E+10	1.8E+10	1.7E+10
90%	314.2	1.9E+22	7.3E+22	4.0E+23	5.4E+21	2.3E+21	5.5E+21	4.3E+21	1.2E+22	1.1E+22	1.0E+22

Table S2. Kinetic parameters derived from the use of the OFW and KAS approaches for different reactions model – Wood + NaCl.

**OFW**											
**α**	**$E_{a,\alpha}$ (kJ/mol)**	**Pre-exponential factor (A_α, s^{-1})**									
		**F1**	**F2**	**F3**	**D2**	**D3**	**R2**	**R3**	**A2**	**A3**	**A4**
10%	125.4	1.5E+09	1.5E+09	1.6E+09	7.1E+07	1.6E+07	7.1E+08	4.8E+08	4.5E+09	6.5E+09	7.9E+09
20%	134.0	6.2E+09	6.9E+09	7.8E+09	5.9E+08	1.4E+08	2.9E+09	2.0E+09	1.3E+10	1.7E+10	1.9E+10
30%	140.0	1.3E+10	1.6E+10	2.0E+10	1.9E+09	4.7E+08	6.1E+09	4.2E+09	2.2E+10	2.7E+10	2.9E+10
40%	139.0	7.8E+09	1.0E+10	1.3E+10	1.4E+09	3.7E+08	3.4E+09	2.4E+09	1.1E+10	1.2E+10	1.3E+10
50%	137.3	4.8E+09	6.9E+09	1.0E+10	1.1E+09	2.9E+08	2.0E+09	1.4E+09	5.8E+09	6.1E+09	6.3E+09
60%	136.8	4.2E+09	7.0E+09	1.2E+10	1.1E+09	3.2E+08	1.7E+09	1.2E+09	4.4E+09	4.5E+09	4.5E+09
70%	137.2	4.7E+09	9.1E+09	2.0E+10	1.3E+09	4.2E+08	1.8E+09	1.3E+09	4.3E+09	4.1E+09	4.1E+09
80%	155.0	1.3E+11	3.1E+11	9.3E+11	3.7E+10	1.3E+10	4.3E+10	3.2E+10	9.9E+10	9.1E+10	8.8E+10
90%	239.0	2.6E+16	1.0E+17	5.7E+17	7.7E+15	3.3E+15	7.8E+15	6.1E+15	1.7E+16	1.5E+16	1.4E+16
**KAS**											
**α**	**$E_{a,\alpha}$ (kJ/mol)**	**Pre-exponential factor (A_α, s^{-1})**									
		**F1**	**F2**	**F3**	**D2**	**D3**	**R2**	**R3**	**A2**	**A3**	**A4**
10%	122.9	7.2E+08	7.6E+08	8.0E+08	3.5E+07	8.1E+06	3.5E+08	2.3E+08	2.2E+09	3.2E+09	3.9E+09
20%	131.6	3.2E+09	3.6E+09	4.0E+09	3.1E+08	7.4E+07	1.5E+09	1.0E+09	6.8E+09	8.7E+09	9.8E+09
30%	137.6	7.1E+09	8.6E+09	1.0E+10	1.0E+09	2.5E+08	3.3E+09	2.2E+09	1.2E+10	1.4E+10	1.5E+10
40%	136.3	3.9E+09	5.1E+09	6.8E+09	7.1E+08	1.9E+08	1.7E+09	1.2E+09	5.4E+09	6.1E+09	6.4E+09
50%	134.3	2.3E+09	3.3E+09	4.9E+09	5.0E+08	1.4E+08	9.6E+08	6.7E+08	2.7E+09	2.9E+09	3.0E+09
60%	133.7	2.0E+09	3.2E+09	5.6E+09	5.0E+08	1.5E+08	7.8E+08	5.6E+08	2.0E+09	2.1E+09	2.1E+09
70%	134.1	2.1E+09	4.1E+09	8.9E+09	6.0E+08	1.9E+08	8.0E+08	5.8E+08	1.9E+09	1.9E+09	1.8E+09
80%	152.6	7.1E+10	1.8E+11	5.3E+11	2.1E+10	7.6E+09	2.4E+10	1.8E+10	5.6E+10	5.1E+10	4.9E+10
90%	239.9	3.0E+16	1.2E+17	6.4E+17	8.7E+15	3.7E+15	8.8E+15	6.9E+15	2.0E+16	1.7E+16	1.6E+16

Table S3. Kinetic parameters derived from the use of the OFW and KAS approaches for different reactions model – Wood + KCl.

OFW											
α	$E_{a,\alpha}$ (kJ/mol)	Pre-exponential factor (A_α, s^{-1})									
		F1	F2	F3	D2	D3	R2	R3	A2	A3	A4
10%	128.6	4.8E+09	5.0E+09	5.3E+09	2.3E+08	5.4E+07	2.3E+09	1.6E+09	1.5E+10	2.1E+10	2.6E+10
20%	135.1	1.2E+10	1.3E+10	1.5E+10	1.2E+09	2.8E+08	5.7E+09	3.9E+09	2.5E+10	3.3E+10	3.7E+10
30%	138.2	1.3E+10	1.6E+10	1.9E+10	1.9E+09	4.6E+08	6.0E+09	4.1E+09	2.2E+10	2.6E+10	2.8E+10
40%	137.1	7.1E+09	9.2E+09	1.2E+10	1.3E+09	3.4E+08	3.1E+09	2.2E+09	9.9E+09	1.1E+10	1.2E+10
50%	136.3	5.2E+09	7.5E+09	1.1E+10	1.2E+09	3.2E+08	2.2E+09	1.6E+09	6.3E+09	6.7E+09	6.9E+09
60%	136.3	5.1E+09	8.3E+09	1.5E+10	1.3E+09	3.8E+08	2.0E+09	1.5E+09	5.3E+09	5.4E+09	5.4E+09
70%	137.3	6.1E+09	1.2E+10	2.6E+10	1.7E+09	5.5E+08	2.3E+09	1.7E+09	5.6E+09	5.4E+09	5.3E+09
80%	162.1	5.9E+11	1.5E+12	4.4E+12	1.8E+11	6.4E+10	2.0E+11	1.5E+11	4.7E+11	4.3E+11	4.2E+11
90%	248.5	1.2E+17	4.7E+17	2.6E+18	3.5E+16	1.5E+16	3.6E+16	2.8E+16	7.9E+16	6.9E+16	6.4E+16
KAS											
α	$E_{a,\alpha}$ (kJ/mol)	Pre-exponential factor (A_α, s^{-1})									
		F1	F2	F3	D2	D3	R2	R3	A2	A3	A4
10%	126.3	2.6E+09	2.7E+09	2.9E+09	1.3E+08	2.9E+07	1.2E+09	8.4E+08	7.9E+09	1.1E+10	1.4E+10
20%	132.8	6.5E+09	7.3E+09	8.2E+09	6.3E+08	1.5E+08	3.1E+09	2.1E+09	1.4E+10	1.8E+10	2.0E+10
30%	135.8	7.0E+09	8.4E+09	1.0E+10	9.9E+08	2.5E+08	3.2E+09	2.2E+09	1.2E+10	1.4E+10	1.5E+10
40%	134.3	3.5E+09	4.6E+09	6.1E+09	6.4E+08	1.7E+08	1.5E+09	1.1E+09	4.9E+09	5.5E+09	5.8E+09
50%	133.4	2.5E+09	3.6E+09	5.4E+09	5.5E+08	1.5E+08	1.0E+09	7.4E+08	3.0E+09	3.2E+09	3.3E+09
60%	133.3	2.3E+09	3.8E+09	6.6E+09	5.9E+08	1.7E+08	9.2E+08	6.6E+08	2.4E+09	2.4E+09	2.5E+09
70%	134.3	2.8E+09	5.5E+09	1.2E+10	7.9E+08	2.6E+08	1.1E+09	7.8E+08	2.6E+09	2.5E+09	2.5E+09
80%	160.1	3.7E+11	9.2E+11	2.8E+12	1.1E+11	4.0E+10	1.3E+11	9.6E+10	2.9E+11	2.7E+11	2.6E+11
90%	249.9	1.5E+17	5.7E+17	3.1E+18	4.2E+16	1.8E+16	4.3E+16	3.4E+16	9.6E+16	8.3E+16	7.8E+16

Table S4. Kinetic parameters derived from the use of the OFW and KAS approaches for different reaction models – $MgCl_2$.

OFW											
α	$E_{a,\alpha}$ (kJ/mol)	Pre-exponential factor (A_α, s^{-1})									
		F1	F2	F3	D2	D3	R2	R3	A2	A3	A4
10%	134.4	1.4E+11	1.5E+11	1.6E+11	7.0E+09	1.6E+09	6.9E+10	4.7E+10	4.4E+11	6.4E+11	7.7E+11
20%	161.0	1.7E+13	1.9E+13	2.1E+13	1.6E+12	3.8E+11	7.8E+12	5.3E+12	3.5E+13	4.5E+13	5.1E+13
30%	147.6	1.8E+11	2.1E+11	2.6E+11	2.5E+10	6.3E+09	8.1E+10	5.6E+10	3.0E+11	3.5E+11	3.8E+11
40%	146.9	4.6E+10	6.1E+10	8.1E+10	8.5E+09	2.2E+09	2.0E+10	1.4E+10	6.5E+10	7.3E+10	7.7E+10
50%	152.3	7.5E+10	1.1E+11	1.6E+11	1.7E+10	4.6E+09	3.2E+10	2.2E+10	9.0E+10	9.6E+10	9.9E+10
60%	153.2	7.0E+10	1.1E+11	2.0E+11	1.8E+10	5.3E+09	2.8E+10	2.0E+10	7.3E+10	7.4E+10	7.5E+10
70%	152.7	5.3E+10	1.0E+11	2.2E+11	1.5E+10	4.8E+09	2.0E+10	1.4E+10	4.8E+10	4.7E+10	4.6E+10
80%	171.2	8.5E+11	2.1E+12	6.3E+12	2.5E+11	9.1E+10	2.9E+11	2.2E+11	6.7E+11	6.2E+11	5.9E+11
90%	224.7	1.8E+14	7.2E+14	4.0E+15	5.4E+13	2.3E+13	5.5E+13	4.3E+13	1.2E+14	1.1E+14	9.8E+13
KAS											
α	$E_{a,\alpha}$ (kJ/mol)	Pre-exponential factor (A_α, s^{-1})									
		F1	F2	F3	D2	D3	R2	R3	A2	A3	A4
10%	133.0	9.5E+10	1.0E+11	1.1E+11	4.7E+09	1.1E+09	4.6E+10	3.1E+10	2.9E+11	4.3E+11	5.2E+11
20%	160.5	1.4E+13	1.6E+13	1.8E+13	1.4E+12	3.3E+11	6.7E+12	4.6E+12	3.0E+13	3.9E+13	4.4E+13
30%	145.9	1.1E+11	1.4E+11	1.6E+11	1.6E+10	4.0E+09	5.2E+10	3.5E+10	1.9E+11	2.2E+11	2.4E+11
40%	144.7	2.6E+10	3.5E+10	4.6E+10	4.8E+09	1.3E+09	1.2E+10	8.1E+09	3.7E+10	4.1E+10	4.4E+10
50%	150.1	4.3E+10	6.3E+10	9.4E+10	9.6E+09	2.7E+09	1.8E+10	1.3E+10	5.2E+10	5.5E+10	5.7E+10
60%	150.8	3.9E+10	6.5E+10	1.1E+11	1.0E+10	3.0E+09	1.6E+10	1.1E+10	4.1E+10	4.2E+10	4.2E+10
70%	150.1	2.9E+10	5.5E+10	1.2E+11	8.0E+09	2.6E+09	1.1E+10	7.8E+09	2.6E+10	2.5E+10	2.5E+10
80%	169.2	5.4E+11	1.3E+12	4.0E+12	1.6E+11	5.8E+10	1.9E+11	1.4E+11	4.3E+11	3.9E+11	3.8E+11
90%	223.9	1.6E+14	6.1E+14	3.4E+15	4.5E+13	1.9E+13	4.6E+13	3.6E+13	1.0E+14	8.9E+13	8.3E+13

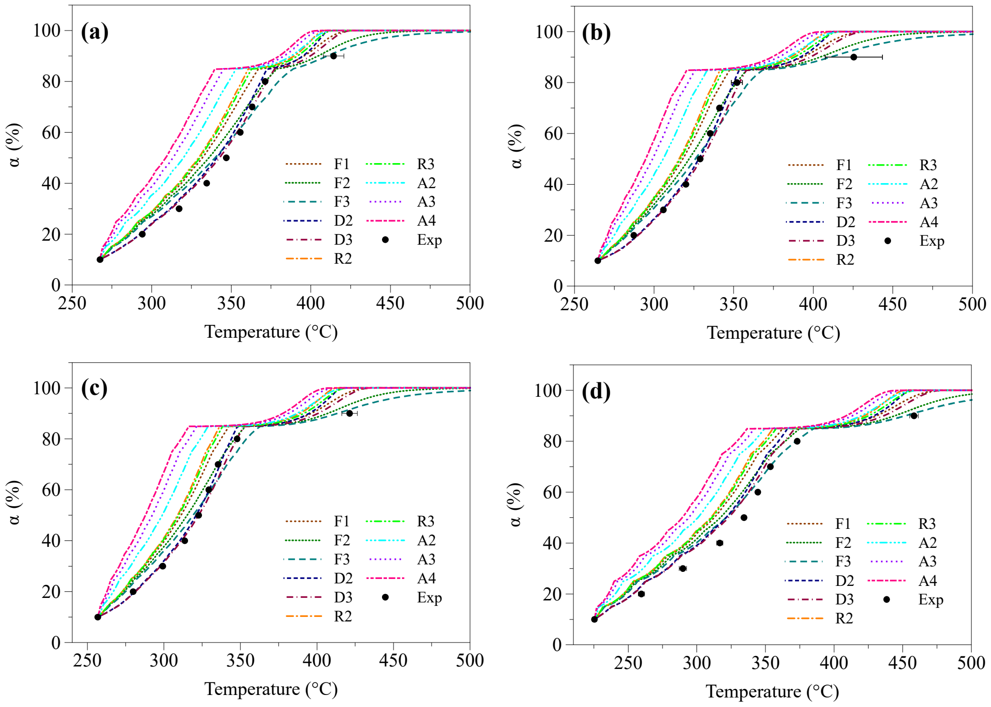

Figure S1. Evolution of α as a function of the temperature for a heating rate of 10 °C/min in the case of (**a**) wood and samples impregnated with (**b**) NaCl, (**c**) KCl and (**d**) $MgCl_2$; comparison of experimental data (noted 'Exp') with predicted ones obtained from the use of the OFW model integrating 10 different reaction mechanisms.

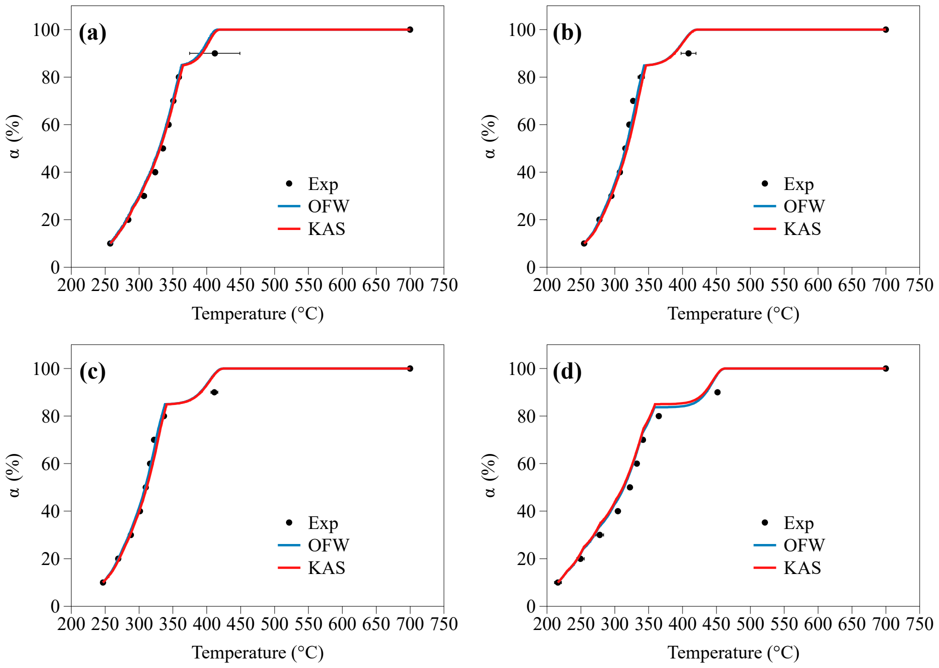

Figure S2. Evolution of α as a function of the temperature for a heating rate of 5 °C/min in the case of (**a**) wood and samples impregnated with (**b**) NaCl, (**c**) KCl and (**d**) $MgCl_2$; comparison of experimental data (noted 'Exp') with predicted ones obtained from the use of the OFW, KAS models integrating the D3 reaction mechanism.

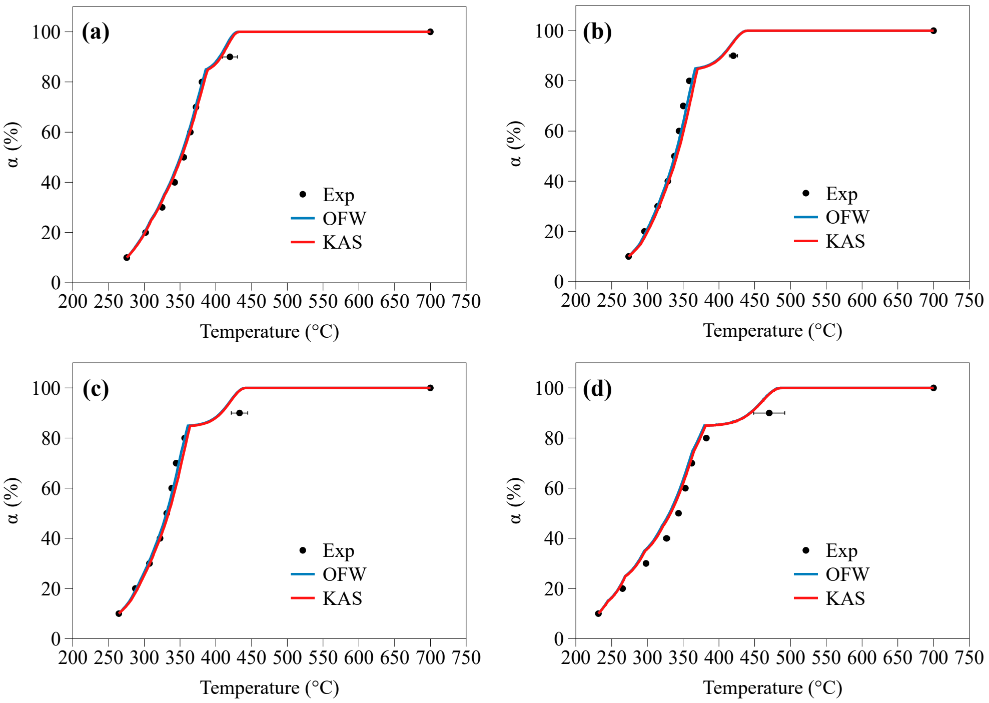

Figure S3. Evolution of α as a function of the temperature for a heating rate of 15 °C/min in the case of (**a**) wood and samples impregnated with (**b**) NaCl, (**c**) KCl and (**d**) $MgCl_2$; comparison of experimental data (noted 'Exp') with predicted ones obtained from the use of the OFW, KAS models integrating the D3 reaction mechanism.

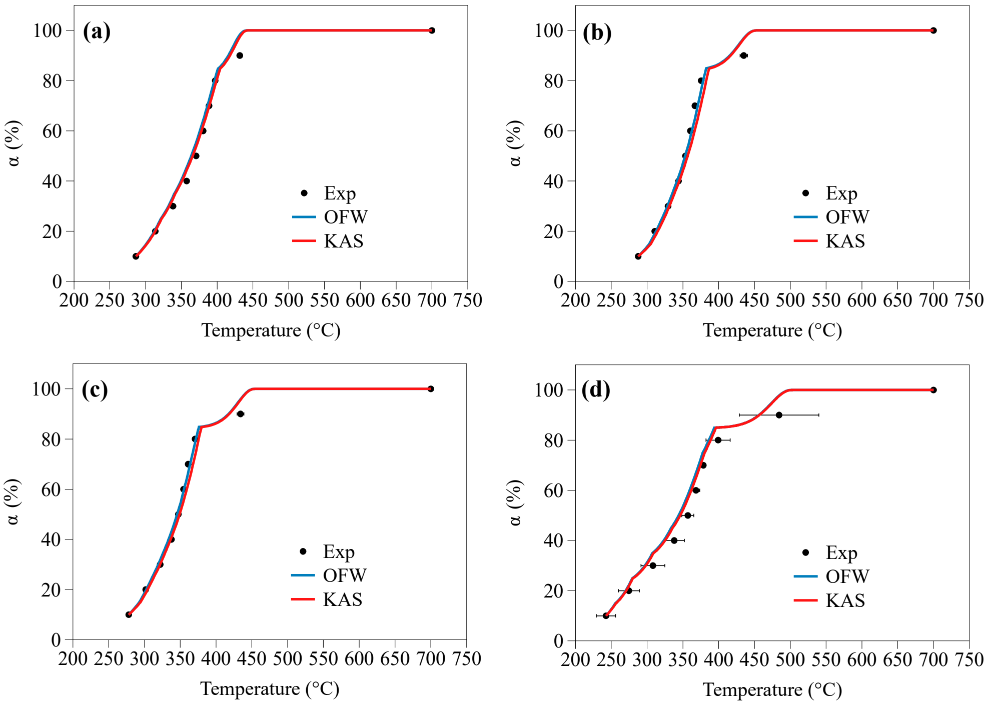

Figure S4. Evolution of α as a function of the temperature for a heating rate of 30 °C/min in the case of (**a**) wood and samples impregnated with (**b**) NaCl, (**c**) KCl and (**d**) $MgCl_2$; comparison of experimental data (noted 'Exp') with predicted ones obtained from the use of the OFW, KAS models integrating the D3 reaction mechanism.
